# Evaluation of Bacterial Composition and Viability of Equine Feces after Processing for Transplantation

**DOI:** 10.3390/microorganisms11020231

**Published:** 2023-01-17

**Authors:** Clémence Loublier, Bernard Taminiau, Julia Heinen, Laureline Lecoq, Hélène Amory, Georges Daube, Carla Cesarini

**Affiliations:** 1 Equine Clinical Department, Faculty of Veterinary Medicine, University of Liège, Bât. B41, 4000 Liège, Belgium; 2 Fundamental and Applied Research for Animals & Health (FARAH), Faculty of Veterinary Medicine, University of Liège, 4000 Liège, Belgium; 3 Department of Food Sciences—Microbiology, Faculty of Veterinary Medicine, University of Liege, Avenue de Cureghem 10, Bât. B43b, 4000 Liège, Belgium

**Keywords:** horse, microbiota, transfaunation, bacteria, fecal, propidium monoazide

## Abstract

Fecal microbiota transplantation (FMT) has been used empirically for decades in equine medicine to treat intestinal dysbiosis but evidence-based information is scarce. This in vitro study aimed at assessing the effect of a commonly used pre-FMT processing method on the bacterial composition and viability of the fecal filtrate. Three samples of fresh equine manure (T_0_) were processed identically: the initial manure was mixed with 1 L of lukewarm water and chopped using an immersion blender to obtain a mixture (T_1_), which was left uncovered during 30 min (T_2_) and percolated through a sieve to obtain a fecal filtrate (T_3_). Samples were taken throughout the procedure (Tn) and immediately stored at 4 °C until processing. The 16S rDNA amplicon profiling associated with propidium monoazide treatment was performed on each sample to select live bacteria. Analyses of α and β diversity and main bacterial populations and quantitative (qPCR) analysis were performed and statistically compared (significance *p* < 0.05) between time points (T_0_–T_3_). No significant differences in ecological indices or mean estimated total living bacteria were found in the final fecal filtrate (T_3_) in regard to the original manure (T_0_); however, relative abundances of some minor genera (*Fibrobacter*, WCHB1-41_ge and *Akkermansia*) were significantly different in the final filtrate. In conclusion, the results support the viability of the major bacterial populations in equine feces when using the described pre-FMT protocol.

## 1. Introduction

Fecal microbiota transplantation (FMT) consists of the infusion of feces from a healthy donor to the gastrointestinal tract of a recipient patient, in order to treat a specific disease associated with an alteration of gut microbiota. In human patients suffering from a recurrent intestinal infection by *Clostridioides difficile*, this therapeutic option has been demonstrated to be efficient [1]. Additional findings suggest that FMT may also play a role in the management of several other disorders associated with alterations of gut microbiota such as ulcerative colitis, metabolic syndrome or autism [1]. Given the promising results of FMT in humans, the clinical efficacy of this approach should also be tested for the prevention and treatment of various diseases associated with dysbiosis in horses [2].

In veterinary medicine, transplantation of rumen content remains a common treatment for multiple gastrointestinal disorders in cattle [3] and historically, equine practitioners have used FMT in horses with diarrhea with the intent of restoring the intestinal microbial balance [4]. However, despite its widespread use in equine medicine, there is no evidence-based standardized protocol of stool processing for FMT. Nowadays, the preparation procedure of FMT in horses is based on extrapolation from the human literature and expert opinions [4,5].

Living microbes are believed to be the therapeutic agent in FMT [6]. The efficacy of FMT is therefore dependent on the ability of live commensal bacteria from the donor to proliferate within the gut recipient [7,8]. Fecal manipulation has been shown to affect bacterial viability in human and other animal samples [9,10]. It has been confirmed in human medical studies that oxygen exposure affects the viability of the fecal microbial content, disproportionately reducing the abundance of anaerobic bacteria and their capacity to produce important anti-inflammatory metabolites [11,12,13,14,15]. More specifically, sample blending was directly associated with detrimental consequences on oxygen-sensitive bacterial species. For this reason, some authors advocate for the use of manual homogenization for FMT processing in human medicine [12].

The ability to accurately assess the bacterial viability of donor fecal samples is critical for developing useful and standardized protocols for the preparation of FMT material. Previous efforts to assess the microbial viability of fecal material used for FMT have been limited. Some authors have used culture methods, which isolate only a small subset of the total microbiota [13,16]. Others, despite using more selective high sequencing methods, were still unable to distinguish the deoxyribonucleic acid (DNA) of viable from non-viable cells or extracellular DNA [17]. A potentially effective strategy to overcoming these challenges is to combine quantitative polymerase chain reaction (qPCR) with propidium monoazide (PMA) treatment [11,18]. PMA is a fluorescent DNA-binding dye that is unable to cross the intact membrane of a viable cell. When the cell membrane is compromised, PMA enters the cytoplasm and intercalates into DNA. Any DNA bounded by PMA is permanently modified through photolysis and its exposure to photons renders it thereby not amplifiable by PCR [19].

To our knowledge, no studies have been performed to assess the effects of this manipulation method on equine fecal bacterial populations before FMT. The objective of this in vitro study was to evaluate the effects of the different steps of a common pre-FMT protocol in healthy horse manure on the bacterial composition, quantification and survival of the final fecal filtrate. We hypothesized that aggressive fiber mixing with an immersion blender in combination with oxygen exposure would cause a significant reduction in the abundance and viability of the anaerobic bacteria.

## 2. Materials and Methods

### 2.1. Study Design and Sample Collection

#### 2.1.1. Donor Horse

Approximately 1 kg of freshly passed feces was collected directly from the rectal ampulla of a healthy donor horse belonging to the teaching herd of the University of Liege. The donor horse was a 19-year-old Polish warmblood mare kept in a stall on straw bedding, fed a diet of 100% haylage (square bales, 60% dry matter, provided at 1.5% of its body weight in two meals per day), with ad libitum access to water. To mimic optimal clinical conditions, the donor horse was chosen based on a medical history free of clinical infectious disease and antimicrobial or anti-inflammatory treatment for the last 6 months, normal physical examination, and up-to-date deworming and vaccination protocols. A specific diagnostic panel detection for fecal pathogens (quantitative fecal egg count with McMaster technique, culture for *Clostridioides difficile*, ELISA for toxins A and B and *Clostridium perfringens* enterotoxin, and PCR for detecting *Salmonella* spp.) was performed on the feces from the donor horse and confirmed to be negative.

#### 2.1.2. Preparations before Carrying out the Procedure

In order to reduce the potential for external contamination with bacterial DNA, objects intended to be in direct contact with feces during the experimental procedure were cleaned and disinfected the day before. On the day of the study, just before starting any procedure, all buckets were flushed with 20 to 40 mL of sterile NaCl 0.9%. A 2 mL aliquot was collected and kept frozen at −20 °C to assess potential contamination in the case of aberrant results. Researchers directly manipulating fecal material during the procedure wore sterile gloves, in order to minimize external bacterial contamination.

Fecal material coming from a single rectal content was divided within 10 min of collection in three fecal subsamples (A, B, C) of 300 g by using a precision electronic scale (Avantor delivered by VWR, series: LA, LPW, LP, LPC), each being placed in an individual plastic bucket. To assess the repeatability of the procedure, each fecal subsample was handled individually, one after the other, and following the same successive processing steps. Different buckets were used to process each fecal subsample. The immersion blender and sieve were rinsed abundantly with tap water until macroscopically clean before each use.

#### 2.1.3. Sample Processing, Collection and Storage

Stools were processed and sampled as follows: at first, fecal material was sampled prior to any manipulation (T_0_). As described by Stewart and collaborators [20], the core of a fecal ball was sampled in order to avoid external bacterial contamination. Five grams of fecal material were placed in a 30 mL irradiated container with a spoon (Boettger, Ref. 07-043-3107, Bodenmais/Germany), approximately half of whose volume contained the fecal sample and half of air.

Then, 1 L of lukewarm tap water (at approximately 28 °C) was added to each bucket containing 300 g of fresh feces. Water temperature was measured immediately before use by means of an infrared thermometer (model: RAK FI03, Crosne, France). Feces and water were mixed for 2 min by using an immersion blender (model: Bosch, Bruxelles, 200–240 V; 50–60 Hz; 600 W; 11,500 rpm). Samples were taken from the resulting mixture (T_1_). Then, the fecal mixture was left uncovered in the bucket at room temperature for 30 min and sampled again (T_2_). Finally, the fecal mixture was filtered through a sieve (model: number 704,225, stainless steel, Gipuzkoa, Spain, 25 × 25 × 10 cm, 181.43 g, 0.45 mm hole diameter) and the final fecal filtrate, ready for a potential FMT, was sampled (T_3_).

**Figure 1:**
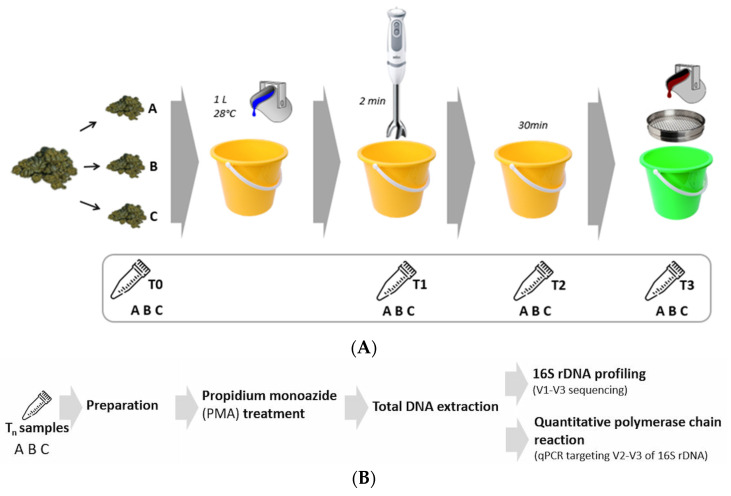
(**A**) Schematic workflow showing the different steps of the protocol used in this study to prepare equine feces for transplantation and the sampling points (Tn) for each subsample (A, B, C) along the procedure. Initial manure (T_0_) was mixed with 1 L of lukewarm water and chopped for 2 min using an immersion blender to obtain a mixture (T_1_), which was left uncovered during 30 min (T_2_) and percolated through a sieve to obtain a fecal filtrate (T_3_). (**B**) Diagram describing the analyses performed on each sample taken in duplicate at different time points (Tn = T_0_, T_1_, T_2_, T_3_) during the fecal manipulation procedure for each subsample (A, B, C).

An illustration depicting the different steps of the fecal processing and sampling is shown in Figure 1A. All samples were taken in duplicate (2 aliquots) with a sterile syringe (15 mL) and placed in the previously described 30 mL irradiated containers, approximately half of whose volume contained the fecal sample and half of air. Samples were immediately refrigerated at 4 °C for less than 6 h until analysis. A diagram showing the different analyses performed on each sample is shown in Figure 1B.

### 2.2. Propidium Monoazide (PMA) Treatment

For the PMA-treated aliquots, we proceeded as follows: for solid samples (T_0_), 200 g of stool were homogenized with 500 μL of saline water 0.9% before adding 2 μL of 20 mM PMA dye (Biotium, Fremont, CA, USA). We incubated them at room temperature for 10 min in the dark, vortexing for several seconds every 2 min. We then photolyzed the aliquots under an LED light (PMA-Lite LED photolysis device) for 15 min, rotating them every 3 min. After photolysis, we centrifuged them for 2 min at 10,000 rpm and we eliminated the supernatant before extraction. For samples T_1_, T_2_, T_3_, which were not solid (fecal mixture or filtrate), a slightly different protocol was performed. Indeed, 1.5 mL of the mixture/filtrate sample was homogenized with 500 μL of saline water 0.9% before adding 1.25 μL of PMA to the aliquot. We incubated them at room temperature for 5 min in the dark, vortexing for several seconds every minute. We then photolyzed the aliquots under an LED light for 10 min. After photolysis, we centrifuged them for 2 min at 10.000 rpm and we eliminated the supernatant before extraction.

In non-PMA treated aliquots (control), 2 μL of PBS was added instead of PMA. We also ran controls that replaced 2 μL of 20 mM PMA dye with water and underwent the same procedure as for PMA sequencing.

### 2.3. Bacterial DNA Extraction and 16S Amplicon Sequencing

Total bacterial DNA was extracted from each fecal subsample (A, B, C) and at each processing step (T_0–3_). Total bacterial DNA extraction was performed before (aliquot 1) and after PMA treatment (aliquot 2) with the PSP Spin Stool DNA Plus Kit 00310 (Invitek, Berlin, Germany), following the manufacturer’s recommendations.

Polymerase chain reaction (PCR) amplification of the 16S rDNA V1–V3 hypervariable region and library preparation was performed with the following primers (with Illumina overhand adapters): forward (50-GAGAGTTTGATYMTGGCTCAG-30) and reverse (50-ACCGCGGCTGCTGGCAC30). Each PCR product was purified with the Agencourt AMPure XP beads kit (Beckman Coulter, Pasadena, CA, USA) and submitted to a second PCR round for indexing, using the Nextera XT index primers 1 and 2. After purification, PCR products were quantified using the Quant-iT PicoGreen (Thermo Fisher Scientific, Waltham, MA, USA) and diluted to 10 ng/μL. A final quantification of each library was performed using the KAPA SYBR FAST quantitative PCR kit (Kapa Biosystems, Wilmington, MA, USA) before normalization, pooling and sequencing on a MiSeq sequencer using V3 reagents (Illumina, San Diego, CA, USA). Positive controls using DNA from 20 defined bacterial species and a negative control (from the PCR step) were included in the sequencing run [21]. Raw amplicon sequencing libraries were submitted to the NCBI database under BioProject number PRJNA850165.

### 2.4. Sequence Analysis and 16S rDNA Profiling

Sequence read processing was performed as previously described [22] using the MOTHUR software package v142.1 for sequence cleaning, taxonomical assignment and OTU clustering (0.03 distance cut off) [23] and VSEARCH algorithm for chimera detection [24]. Sixteen S reference alignments and taxonomical assignments from phylum to genus were performed with MOTHUR and were based upon the SILVA database (v1.38) of full-length 16S rRNA sequences (Silva ribosomal RNA).

To assess the effects of the fecal manipulation procedure on bacterial populations, datasets of 10,000 reads per sample were obtained with MOTHUR and used to evaluate ecological indicators and β diversity.

In order to check the reliability of PMA microbiota method targeting cell DNA, we compared the taxonomic compositions in 16S rDNA-based PMA microbiota with those without PMA using an identical specimen.

### 2.5. Quantitative PCR

The total amount of viable bacteria in each specimen was estimated using qPCR after PMA treatment, as mentioned above. The bacterial load was assessed by a real-time qPCR system (PRISM 7900HT; Applied Biosystems, Foster City, CA, USA) targeting the V2–V3 region of the 16S rDNA as described by Fastres and colleagues [25]. The total bacterial population was assessed by quantifying the number of DNA copies and the results were expressed in base 10 logarithms of gene copies per gram of feces.

Samples were analyzed in a 25 µL reaction mixture consisting of 12.5 µL of SYBR Premix (50 mmol/L KCl, 20 mmol/L Tris-HCl, pH 8.4, 0.2 mmol/L deoxynucleoside triphosphate, 0.625 U TaKaRa Taq (Clontech, Mountain View, CA, USA), 3 mmol/L MgCl_2_, and 10 nmol/L fluorescein), 0.2 mol/L of each primer and 5 µL of DNA. Serial dilutions (100–1000-fold) of extracted DNA were subjected to qPCR with the Uni331F (5′-TCCTACGGGAGGCAGCAGT-3′) and Uni797R (5′GGACTACCAGGGTATCTAATCCTGTT-3′) primers.

### 2.6. Data Analysis

Good’s coverage index and ecological indicators, including the bacterial diversity (inverse Simpson’s index), richness (Chao1 index) and evenness (Simpson’s index-based measure) were calculated with MOTHUR v1.42. The Friedman test in XLSTAT (Addinsoft, Paris, France) was used to analyze the variance between the measures (3 aliquots of 4 time points: T_0_, T_1_, T_2_, T_3_) of the different ecological indices.

Β diversity was visualized with a Bray–Curtis dissimilarity matrix-based non-parametric dimensional scaling (NMDS) model using vegan (https://cran.rproject.org/web/packages/vegan/index.html (accessed on 11 January 2021)) in R. Differences were considered significant with a *p*-value < 0.05. Analysis of molecular variance (AMOVA) and homogeneity of molecular variance (HOMOVA) tests were performed using MOTHUR (10,000 iterations on the subsampled table).

Differential abundances of the bacterial populations between groups were assessed with DESeq, using the Deseq2 v1.32.0 package in R [26].

The viability rate was calculated by dividing the viable cells amplified in the PMA-treated samples over the total number of cells amplified in the non-PMA treated samples. The total amount of viable bacteria was determined using PMA-qPCR and ANOVA analyses were performed and followed by paired post hoc tests corrected with the two-stage linear step-up procedure of Benjamini, Krieger and Yekutieli using PRISM 7 (GraphPad Software, San Diego, CA, USA) to analyze the statistical differences between time points. Differences were considered significant with a *p*-value or a *q*-value < 0.05.

## 3. Results

An average of 6,414,573 reads was first obtained, then 5,544,534 reads after cleaning with a median read length of 490 bp and 3,383,883 reads after chimera removal for the total study. A data table was built containing 206 final phylotypes (at the genus level) and a mean sampling Good’s coverage of 99.76%, with no statistical difference between groups.

The following results were obtained from the analysis of PMA-treated aliquots (intended to represent viable bacterial populations). Results concerning untreated aliquots can be found in the Supplementary Materials section.

### 3.1. Impact of the Fecal Manipulation Procedure on Fecal Composition

#### 3.1.1. Alpha Diversity

**Figure 2:**
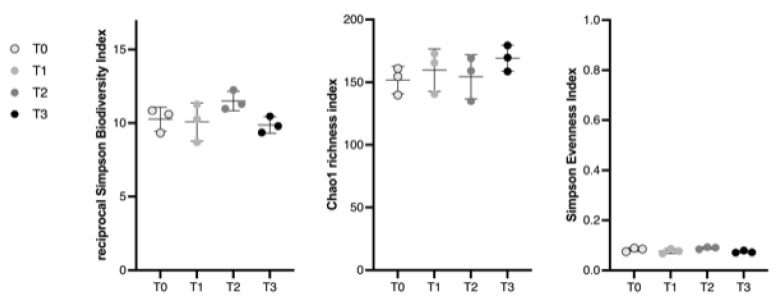
Scatterplots depicting α diversity indices at each step of the fecal manipulation procedure (T_0–3_). Each dot represents a subsample (A, B, C). Bacterial intrinsic diversity was deduced from the reciprocal Simpson biodiversity index, bacterial genus richness from the Chao1 index and bacterial genus evenness from Simpson’s index. No significant difference was found between groups regarding the reciprocal Simpson index, population richness and Simpson-derived evenness based on a Friedman test. Horizontal lines represent the mean and error bars indicate the 95% CIs for each time point.

Microbial population ecological indices of fecal samples were assessed at the genus level. The results of the α diversity, richness and evenness of the bacterial populations throughout the procedure (T_1–2–3_) were not significantly different in regard to the original fecal sample (T_0_) for any of the three subsamples (A, B, C) (Figure 2).

#### 3.1.2. Beta Diversity

**Figure 3:**
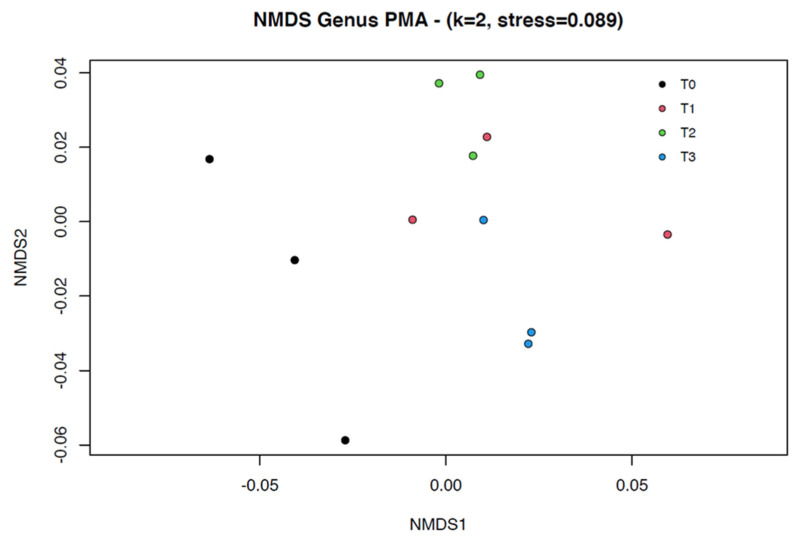
Nonmetric multidimensional scaling (NMDS) model (k = 2, stress = 0.089) based upon a Bray–Curtis dissimilarity matrix of the fecal microbial profiles before (black = fresh feces = T_0_) and at the different steps of the fecal manipulation procedure (red = mixed feces = T1, green = prefiltered feces = T_2_, blue = final fecal filtrate = T_3_). The 3 dots of each color represent the different subsamples (A, B, C). Despite global AMOVA analysis showing a heterogeneous variance between groups regarding β diversity (*p*-value = 0.0008), a paired test did not reveal significant differences. HOMOVA testing yielded non-statistically significant differences.

Group clustering testing did reveal significant differences in variance based on AMOVA analysis (*p*-value = 0.0008). However, paired analysis between the different steps (T_0–3_) did not reveal significant differences. HOMOVA testing yielded non-statistically significant differences (*p*-value = 0.0901). β diversity of the fecal microbial profile was represented using a nonmetric multidimensional scaling (NMDS) model (stress = 0.089, two dimensions) which is shown in Figure 3.

#### 3.1.3. Major Bacterial Populations

**Figure 4:**
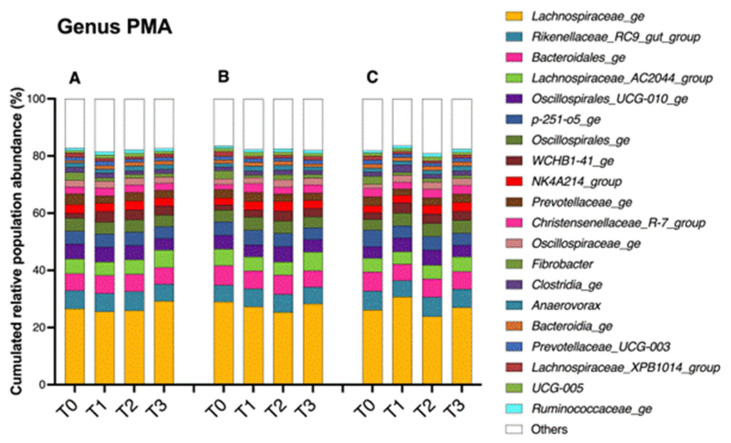
Changes in the bacterial populations of the fecal content assessed by 16S V1–V3 profiling. The bar chart depicts the relative abundances of the main bacterial genera (accounting for more than 1% of the total abundance) present in the fresh feces (T_0_) and in the samples obtained throughout the fecal manipulation procedure (T_1–3_) for each fecal subsample (A, B, C). A total of 206 genera were identified.

A total of 105 families and 206 genera were identified in the fecal subsamples (A, B, C) during the whole fecal manipulation procedure (T_0–1–2–3_). The most abundant bacterial populations were classified in the phyla Firmicutes and Bacteroidota. The most abundant genera were *Lachnospiraceae_ge* (mean relative abundance (RA) 27%), *Rikenellaceae_RC9_gut_group* (mean RA 6%), *Bacteroidales_ge* (mean RA 6%) and *Lachnospiraceae_AC2044_group* (mean RA 5%). The distributions of the main genera and their RAs in the fresh feces (T_0_) and at the different steps of the fecal manipulation procedure (T_1–3_) are depicted in Figure 4.

The proportions of the three bacterial genera differed significantly between T_0_ and T_3_. The genus Fibrobacter was significantly more abundant in the original manure than in the final filtrate (T_0_ = 2.83%, T_3_ = 1.17%; *p*-value < 0.0001). On the other hand, at T_3_, we observed significantly more abundant proportions of the genera WCHB1-41_ge (T_0_ = 1.94%, T_3_ = 3.05%; *p*-value = 0,0037) and Akkermansia (T_0_ = 0.25%, T_3_ = 0.63%; *p*-value = 0,0037).

### 3.2. Impact of the Fecal Manipulation Procedure on Fecal Bacterial Survival

No significant differences were found in the total bacteria results between the steps of the fecal manipulation procedure (T_n_). In the initial fecal sample (T_0_), the mean viability rate of the samples (A, B, C) was 100% and decreased to 96% in the final fecal filtrate (T_3_).

**Figure 5:**
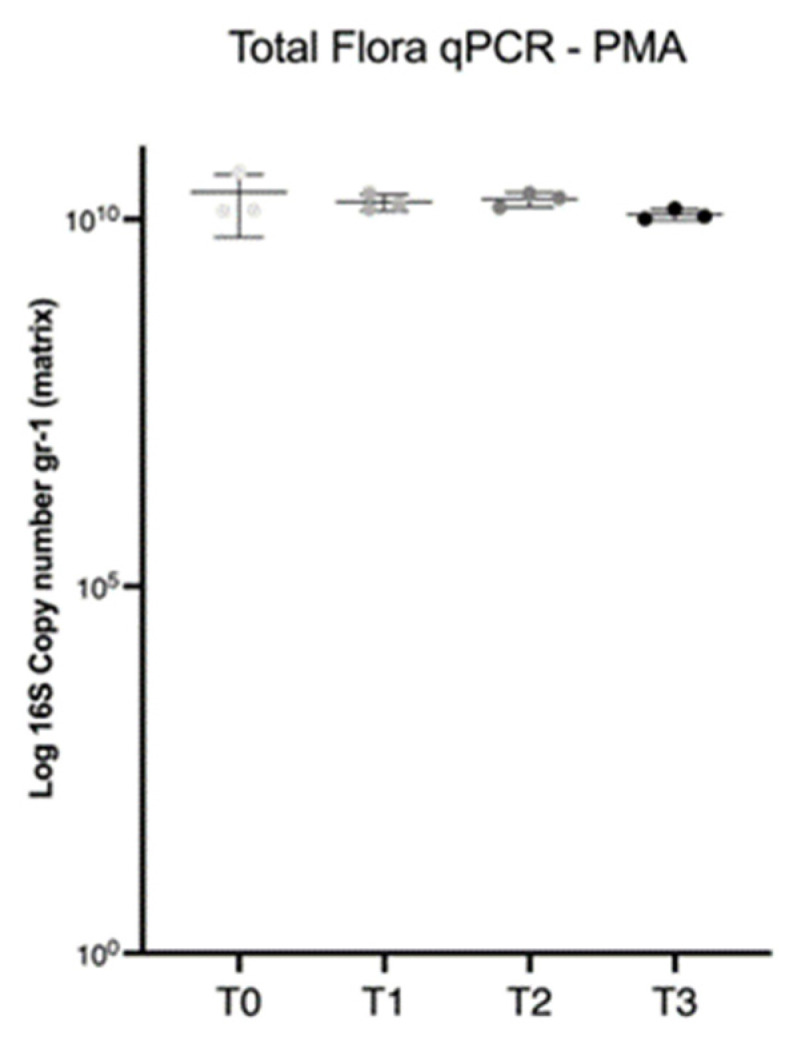
Scatterplot depicting the total bacteria V2–V3 16S estimated by qPCR analysis in the PMA-treated aliquots of the fresh feces (T_0_) and in the fecal filtrate obtained at the different steps of the fecal manipulation procedure (T_1–3_). Each dot represents a subsample (A, B, C). Based on ANOVA analysis, the estimated total bacteria (mean) by the means of qPCR in the final fecal filtrate (T_3_) (1.2 × 10^10^ gene copies/g) were not significantly different from the initial fecal sample (T_0_) (2.3 × 10^10^ gene copies/g).

Based on the ANOVA analysis, the estimated total bacteria (mean) by the means of qPCR in the final fecal filtrate (T_3_) were not significantly different (1.2 × 10^10^ gene copies/g) from the initial fecal sample (T_0_) (2.3 × 10^10^ gene copies/g) (Figure 5).

## 4. Discussion

The efficacy of FMT is dependent on the ability of live beneficial commensal bacteria from the healthy donor to proliferate within the diseased gut recipient [11]. The objective of this study was to evaluate the effects on bacterial composition and survival during the different steps of a commonly used fecal manipulation procedure to obtain a fecal filtrate from healthy horse manure. The results indicate that the described pre-FMT protocol did not seem to induce major modifications of the bacterial population composition and viability regarding the initial fresh feces, supporting its use as a source of live bacteria from a horse donor and a first step towards a successful fecal transplant in the equine species.

Many bacterial populations in the horse gut are obligate or facultative anaerobes, whose viability may depend on how FMT filtrates are prepared, as is the case in humans [12,20]. In the equine species, the phyla Firmicutes, Bacteroidota and Verrucomicrobiota are mentioned as part of the “core equine intestinal microbiota” [27] or the key bacteria present in the GIT of healthy horses [28]. It is crucial to consider that some of these bacteria are presumed to be very sensitive to the presence of oxygen, especially the members of the phylum Firmicutes, while Bacteroidota appears to be more oxygen-tolerant [11].

The pre-FMT protocol used in the present study included the dilution, mixture and filtration of fresh feces, which are the different steps usually performed to prepare equine feces from a donor horse prior to FMT to a recipient horse. This specific method of processing was chosen because it is commonly used in equine practice. A blender was used to homogenize the mixture in an objective and reproducible manner (i.e., in contrast to manual mixing). This could help to better detach bacteria from vegetal fibers but could be more detrimental to oxygen-sensitive species by increasing air flow during high-speed blending. We hypothesized that aggressive fiber mixture with an immersion blender in combination with oxygen exposure would cause the most significant reduction in abundance and viability of anaerobic bacteria. However, the samples taken at different steps of the fecal manipulation procedure (T_1–3_) did not show significant differences in α diversity, main bacterial composition and total live bacteria (estimated by qPCR after PMA treatment) in regard to the original fresh manure (T_0_). Although high-speed blending was used, neither the mixing step (T_1_) nor the oxygen exposure step (T_1–2_) significantly influenced the composition and viability of bacteria in the sample.

This finding differs from what the human literature reports. Using the same viability assessment technique (PMA) as in our study, Papanicolas and collaborators [18] found that ambient air profoundly affected the viable microbial content in human feces, disproportionately reducing the abundance of anaerobic bacteria. In their study, homogenization in ambient air reduced the bacterial viability to 19% in a 30 g fecal sample and the processing of the samples in ambient air resulted in an up to 12-fold reduction in the abundance of important commensal taxa, including the highly butyrogenic species *Faecalibacterium prausnitzii*, *Subdoligranulum variable* and *Eubacterium hallii*.

Some differences that could explain these results are the weight of the fecal sample and the technical characteristics of the blender used. Indeed, in our study, a household immersion blender (model: Bosch 200–240 V; 50–60 Hz; 600 W, 11,500 rpm) was used, which has half of the rotating speed (rpm) of the laboratory blender (model: Waring SS515, 240 V, 22,000 rpm) used in Papanicolas’ study and could have been less aggressive with the fecal sample. Furthermore, given the larger volume of horse feces as compared to humans, our fecal donor sample was 10 times larger than the sample volume used in the human study (300 g vs. 30 g in the previous human study). These differences could have induced a higher air flow exposure and more detrimental mechanical consequences on the anaerobic bacteria in Papanicolas’ study [12].

Although some studies in human medicine advise to use anaerobic conditions for processing FMT, aerobic FMT processing methods could also have a clinical interest. Indeed, the current practices of preparing fecal transplants to treat *C. difficile* infections involve processing under aerobic conditions [29]. Comparisons conducted in recurrent *C. difficile* infected subjects using aerobically and anaerobically prepared FMT filtrates showed similar efficacy [30]. This could be due to the fact that some anaerobic bacterial genera of healthy microbiota produce resilient spores allowing the interindividual transfer of at least a proportion of oxygen-sensitive intestinal bacteria [31]. The aerobic and anaerobic conditions for FMT processing still seem debatable in humans however, with some authors considering that “ambient air processing is the default practice in most clinical trials” [12], whereas the European [1] and British [32] consensus for the FMT guidelines considers that “there appears to be no clear need to process anaerobically, a method which introduces complexity and cost for the treatment of *C. difficile* infections“. However, anaerobic FMT preparation has shown its ability to preserve anaerobes as obligate anaerobes such as *Faecalibacterium prausnitzii*, an anti-inflammatory commensal bacterium whose absence is correlated with active inflammatory bowel diseases (IBD) [11]. Because IBD is typically defined by a lower abundance of anaerobic bacteria [33,34], it is rational to expect that the anaerobic processing of samples would be relevant for FMT success in the treatment of these disorders.

In equine medicine, FMT is commonly prepared under aerobic conditions [4,35].

Concerning β diversity, AMOVA analysis revealed significant differences in the overall variance between the T_0–3_ samples (*p*-value = 0.0008) but paired analysis did not show statistically different results. The procedural steps (water dilution and mixture, oxygen exposure, filtration) could thus somehow affect microbial diversity but the fact that none of the paired analyses was significantly different prevents finding which step was responsible for the overall variance difference. This inability to show the statistical differences in the paired analyses could be due to a low statistical power. Either the number of samples was not big enough to reveal this difference or the variability was too small. However, although the statistical power was weak, drastic changes were not evidenced in the major bacterial populations in association with the fecal manipulation procedures.

The mixture was finally sieved to eliminate the undigested and small particle matter in the fecal mixture and thus avoid any clogging of the nasogastric tube. This step (T_3_) did not alter the main bacterial populations nor their viability. The estimated total bacteria (mean) by means of qPCR in the final fecal filtrate (T_3_) were not significantly different (1.2 × 10^10^ gene copies/g) from the initial fecal sample (T_0_) (2.3 × 10^10^ gene copies/g), indicating that the dilution effect of water did not seem to affect the count of viable bacteria when using the proportions of feces and water described in this study. The use of a sieve rather than a tissue was chosen to optimize the objectivity of the procedure. However, as described previously, a recent equine study has shown good clinical results with the use of a bouffant cap serving as a standard sieve (McKesson 24-inch disposable bouffant caps) to filtrate [35]. Interestingly, despite the total gut bacteria not being significantly altered, the abundances of some minor bacterial populations were significantly different between the original manure (T_0_) and the final fecal filtrate (T_3_). The relative abundance of the genus *Fibrobacter* was significantly lower whereas those of WCHB1-41_ge and *Akkermansia* were significantly higher in the final filtrate. Some of these differences could be explained because of the nature of the sample (solid, fiber-rich vs. filtrated liquid sample). In a study comparing bacterial communities identified in ruminal contents following different sampling methods, the authors found that the relative abundance of the genus *Fibrobacter* was significantly lower in stomach tube samples (liquid) compared with solid samples grabbed though a rumen cannula [36]. An explanation given to justify these findings, that could also make sense in the present study, is the exclusion of fibrous particles in the liquid sample, as *Fibrobacter* is an anaerobic cellulolytic bacterium that facilitates cellulose degradation. In the same study, the bacterial order WCHB1-41 (phylum Kiritimatiellaeota) was enriched in liquid samples (both unstrained and strained through a cheesecloth) as compared to samples directly grabbed though a rumen cannula. Other ruminant studies also found the Kiritimatiellaeota phylum in higher proportion in the liquid compared with the solid phase of the rumen [37,38]. In a recent study on equine FMT to horses with diarrhea, the phylum Kiritimatiellaeota was overrepresented in the healthy samples, which was interpreted as a possible indication of the functional importance of this phylum in the healthy equine gut [35]. No clear explanation was found for the increase in *Akkermansia* in the final fecal filtrate.

The limitations of this study should be considered when interpreting these results. This study has focused solely on bacterial composition, although it is not the only component of the fecal microbiota. The effects of the fecal manipulation procedures described on protozoa, viruses, fungi and other components of intestinal microbiota have not been evaluated and remain unknown. Three different subsamples were taken in order to assess the repeatability of the results. The small sample size could have affected the statistical results, making it more difficult to reveal the potential consequences of manipulation on the composition and viability of the bacteria, although the sample size was consistent with other experimental studies on fecal microbiota [11,12,39]. Another potential limitation lies in the fact that manure was taken from a unique horse donor, which could have affected the results by means of the specific intraindividual resistance or sensitivity of the bacteria against the different steps of the procedure. For example, fecal material from different donors could respond differently to the processing steps, particularly if there is variation in the amount of anaerobes in the community.

The use of PMA for bacterial viability testing in microbiome studies has its limitations. PMA-based viability assays rely on the assumption that all cells with intact membranes are viable without actually proving any metabolic activity. The effectiveness of PMA treatment varies with different bacterial species and in different sample types [40], and it is prone to overestimate the number of live bacteria in a sample [19]. However, the results of the ecological indices

Β diversity, relative abundance and qPCR were similar in the samples treated with PMA and non-treated ones. To avoid protocol variations, all samples (samples T_0–3_ of subsamples A,B,C) were processed simultaneously. However, this means that the T_0_ samples were kept for a longer period (45–60 min), refrigerated at 4 °C, in regard to the T_3_ samples, which could have altered the bacterial viability. Bustamante et al., 2021 revealed that storage of equine fecal samples at room temperature for up to 6 h before freezing had a minimal effect on the fecal microbiota [41]. Furthermore, Burz et al., 2019 showed that refrigeration at 4 °C can be a safe solution to avoid changes in bacterial populations during fecal sample storage for 24 h, which supports the result that the differences in the storage times in our study seem negligible [9]. Finally, the method of analysis could influence the results. Indeed, it has been shown for example that different DNA extraction kits could give different results [42,43]. Thus, it would be interesting to test this protocol with other extraction kits in order to see if this could influence the results of composition and bacterial viability. A future standardization of the methods used in equine microbiota studies seems crucial.

## 5. Conclusions

The results of the present study suggest that despite dilution, oxygen exposure and manipulation prior to transplantation, it is possible to preserve the viability of a high percentage of the equine fecal bacterial load. The variation in the composition, quantity and viability of the fecal bacterial microbiota was not significantly affected either by mixing the fecal sample for 2 min with a domestic immersion blender, by resting it and exposing it to ambient air for 30 min or by filtering the sample using a sieve with a small pore diameter. Although promising, this study should be taken as preliminary data due to the limited sample size coming from a single individual, which precludes further extrapolation to the general equine population. Further research is needed in order to make conclusions about this procedure as a standard for the general equine population.

## Data Availability

Raw amplicon sequencing libraries were submitted to the NCBI database under BioProject number PRJNA850165.

## Supplementary Materials

The following supporting information can be downloaded at: https://www.mdpi.com/article/10.3390/microorganisms11020231/s1, Figure S1: Scatterplots depicting α diversity indices at each step of the fecal manipulation procedure (T_0–3_) without PMA treatment. Each dot represents a subsample (A, B, C). Bacterial intrinsic diversity was deduced from the reciprocal Simpson biodiversity index, bacterial genus richness from the Chao1 index and bacterial genus evenness from Simpson’s index. No significant difference was found between the groups regarding the reciprocal Simpson index, population richness and Simpson-derived evenness based on a Friedman test. Horizontal lines represent the mean and error bars indicate the 95% CIs for each time point. Figure S2: Nonmetric multidimensional scaling (NMDS) model (k = 2, stress = 0.058) based upon a Bray–Curtis dissimilarity matrix of the non-PMA treated fecal microbial profiles before (black = fresh feces = T_0_) and at the different steps of the fecal manipulation procedure (red = mixed feces = T1, green = prefiltered feces = T_2_, blue = final fecal filtrate = T_3_). The 3 dots of each color represent the different subsamples (A, B, C). Despite global AMOVA analysis showing a heterogeneous variance between groups regarding the β diversity (*p*-value = 0.0008), a paired test did not reveal significant differences. HOMOVA testing yielded non-statistically significant differences. Figure S3: Changes in bacterial populations in the non-PMA-treated fecal content assessed by 16S V1–V3 profiling. The bar chart depicts the relative abundance of the main bacterial genera (accounting for more than 1% of the total abundance) present in the fresh feces (T_0_) and in the samples obtained throughout the fecal manipulation procedure (T_1–3_) for each fecal subsample (A, B, C). A total of 192 genera were identified. Figure S4: Scatterplot depicting the total bacteria V2–V3 16S estimated by qPCR analysis in the non-PMA-treated aliquots of the fresh feces (T_0_) and in the fecal filtrate obtained at the different steps of the fecal manipulation procedure (T_1–3_). Each dot represents a subsample (A, B, C). Statistical results based on ANOVA analysis showed significant differences (*p* = 0.07). A paired test has showed significant statistical differences between the steps T_0_–T_1_ (*q*-value = 0.006), T_1_–T_2_ (*q*-value = 0.022) and T_1_–T_3_ (*q*-value = 0.005).
