# Supplementary figures and images for: Evaluation of Bacterial Composition and Viability of Equine Feces after Processing for Transplantation
Source: Microorganisms. 2023 Jan 17;11(2):231. doi: 10.3390/microorganisms11020231 (PMC9966902; doi:10.3390/microorganisms11020231)

● T0  
● T1  
● T2  
● T3

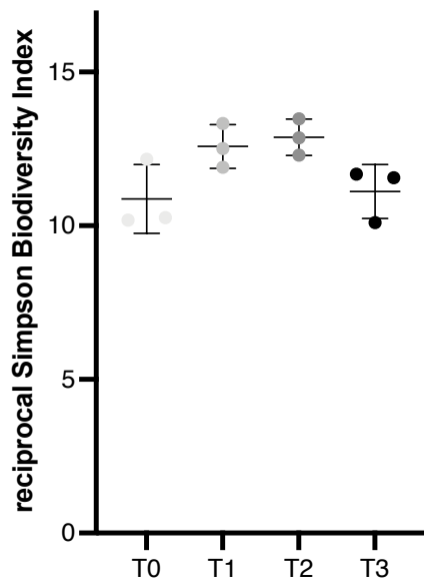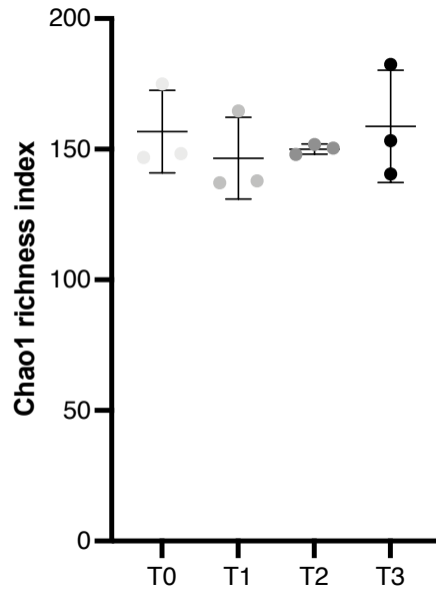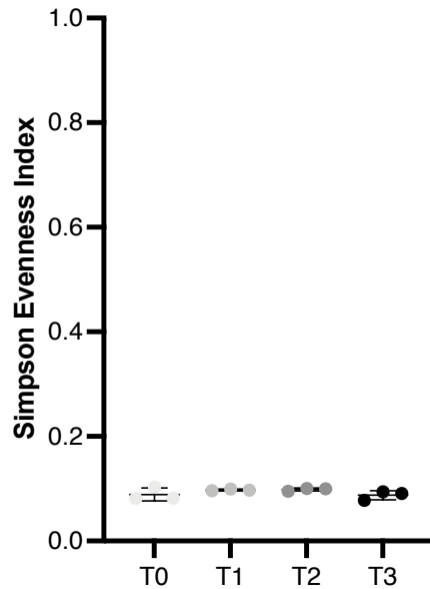

Supplement: Supplementary file 1 [file microorganisms-11-00231-s001.zip › Supplementary data figures/Figure S1 - Alpha nopma.pdf]

NMDS Genus noPMA - (k=2, stress=0.058)

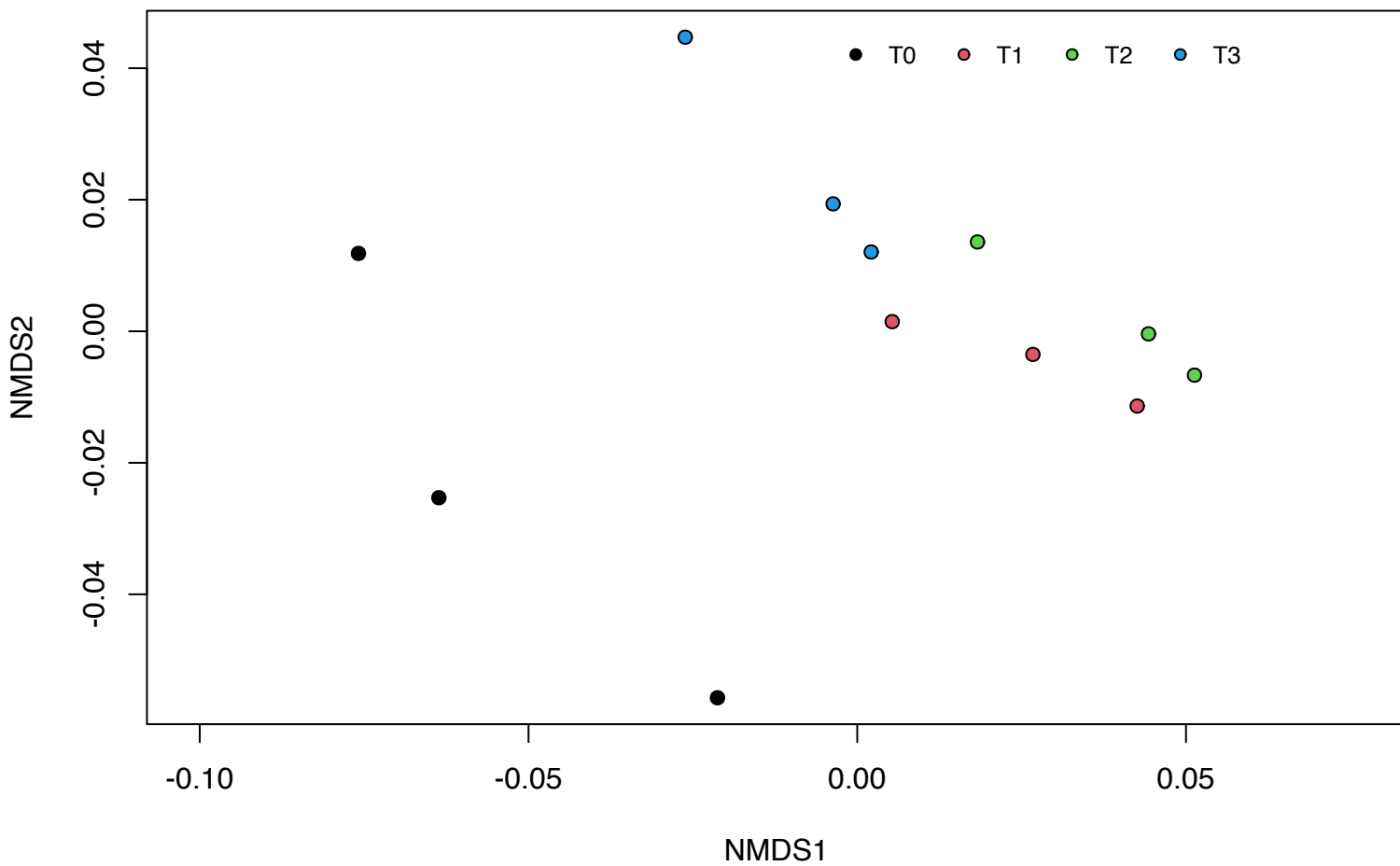

Supplement: Supplementary file 1 [file microorganisms-11-00231-s001.zip › Supplementary data figures/Figure S2 - Beta nopma 2 dimensions.pdf]

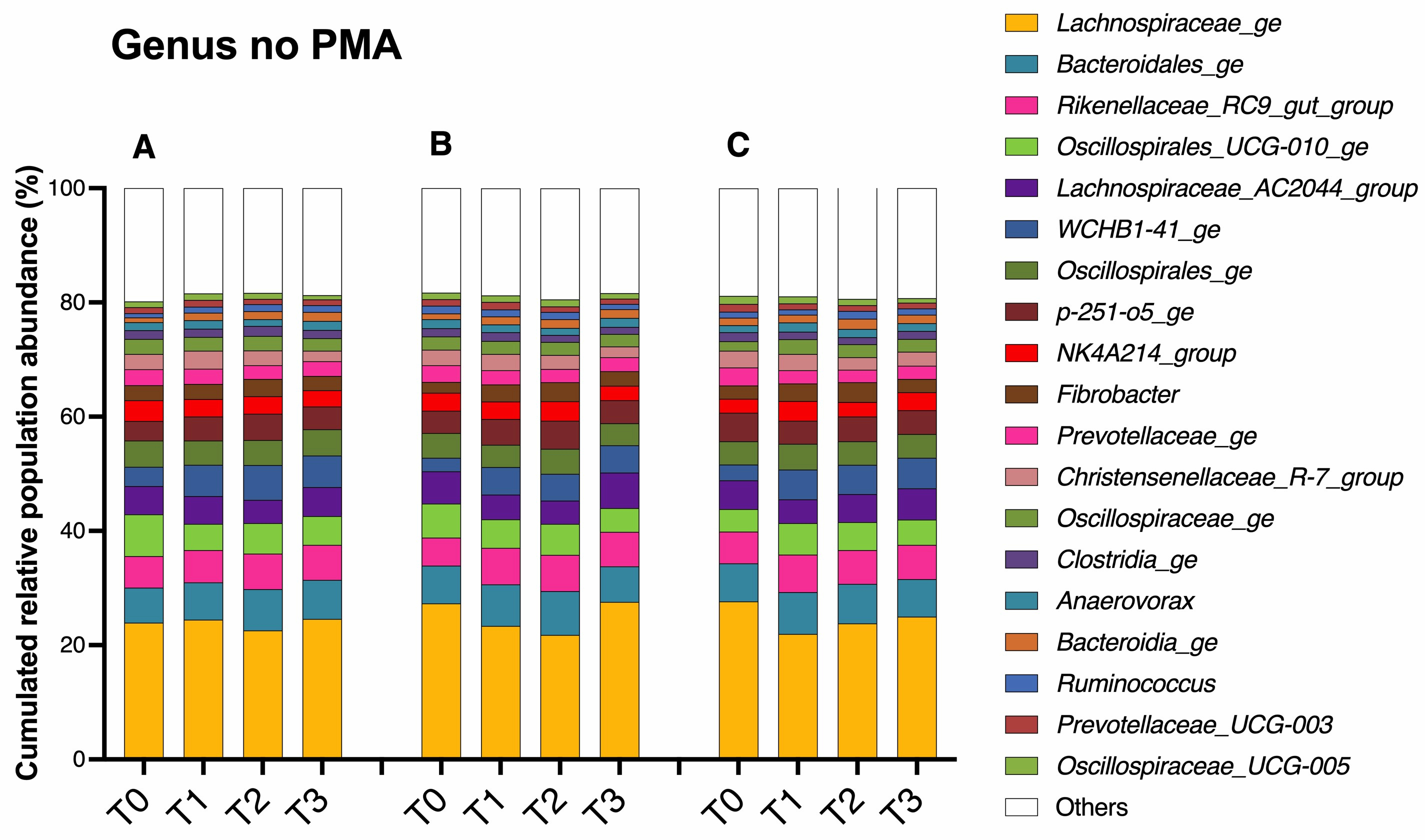

Supplement: Supplementary file 1 [file microorganisms-11-00231-s001.zip › Supplementary data figures/Figure S3 - Genus_abund_noPMA.jpg]
