# Supplementary material for: Evaluation of Bacterial Composition and Viability of Equine Feces after Processing for Transplantation
Source: Microorganisms. 2023 Jan 17;11(2):231. doi: 10.3390/microorganisms11020231 (PMC9966902; doi:10.3390/microorganisms11020231)
Supplement: Supplementary file 1 [file Figure S4 - qPCR_nonlog_T0-T3_nopma.pdf › Supplementary data figures/Figure S4 - qPCR_nonlog_T0-T3_nopma.pdf]

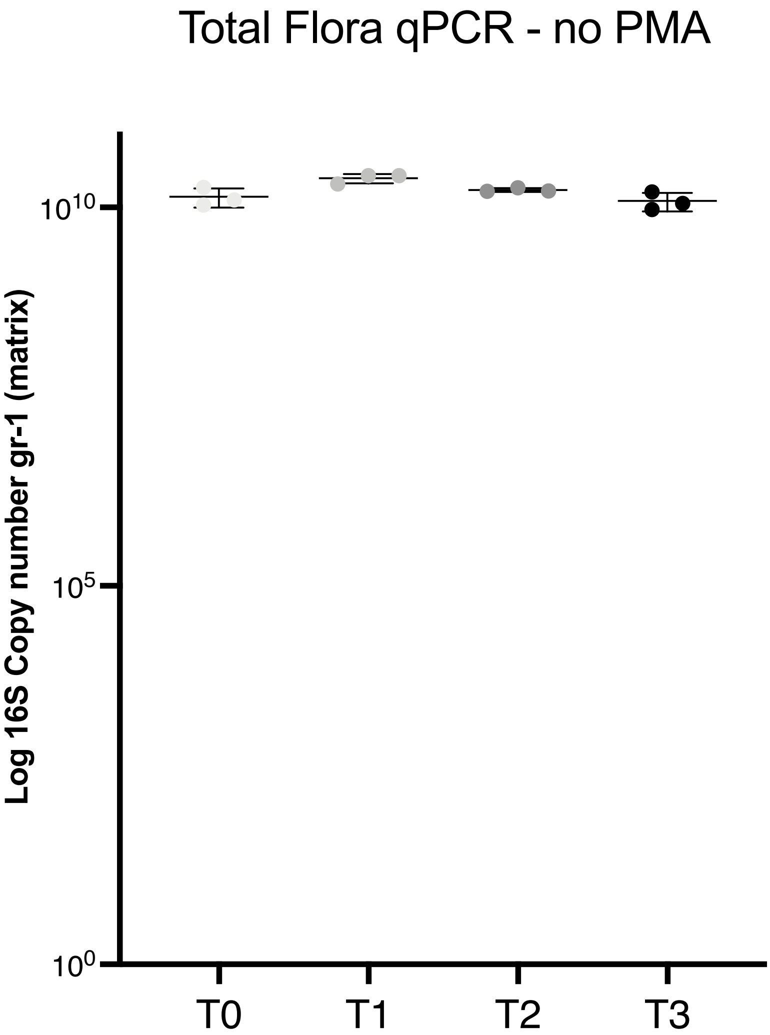
Total Flora qPCR - no PMA
Log 16S Copy number gr-1 (matrix)
10^{10}
10^{5}
10^{0}
T0
T1
T2
T3
